# Towards a radiation-free clinical decision support system for intraoperative spinal alignment assessment

**DOI:** 10.1007/s11517-025-03412-z

**Published:** 2025-07-24

**Authors:** Luis Serrador, Pedro Varanda, Bruno Direito-Santos, Cristina P. Santos

**Affiliations:** 1 Center for MicroElectroMechanical Systems (CMEMS), University of Minho, Largo do Paço, Braga, 4704-553 Portugal; 2 Clinical Academic Center of Braga (2CA-Braga), Hospital of Braga, Lugar de Sete Fontes, S. Victor, Braga, 4710-243 Portugal; 3 Orthopedics Department, Hospital of Braga, Lugar de Sete Fontes, S. Victor, Braga, 4710-243 Portugal

**Keywords:** Spinal surgery, Spinal alignment, Computer-assisted surgery, Clinical decision support system

## Abstract

**Abstract:**

This paper introduces *SpineAlign*, a novel radiation-free clinical decision support system (CDSS) designed to address the challenge of intraoperative spinal alignment assessment during spinal deformity (SD) correction surgeries. *SpineAlign* aims to overcome the current limitations of existing systems by providing a quantitative assessment without radiation exposure in the operating room (OR), thus enhancing the safety and precision of computer-assisted spinal surgeries (CASS). The system focuses on spinal alignment calculation, leveraging Bézier curves and algorithm development to track vertebrae and estimate spinal curvature. Collaborative meetings with clinical experts identified challenges such as patient positioning complexities and limitations of minimal invasiveness. Thus, the method developed involves four algorithms: (1) tracking anatomical planes; (2) estimating the Bézier curve; (3) determining vertebrae positions; and (4) adjusting orientation. A proof of concept (PoC) using a porcine spinal segment validated *SpineAlign*’s integrated algorithms and functionalities. The PoC demonstrated the system’s accuracy and clinical applicability, successfully transitioning a spine without curvature to a lordotic spine. Quantitative evaluation of spinal alignment by the system showed high accuracy, with a maximum root mean squared error of 6$^{\circ }$. The successful PoC marks an initial step towards developing a reliable CDSS for intraoperative spinal alignment assessment without medical image acquisition. Future steps will focus on enhancing system robustness and performing multi-surgeon serial studies to advance *SpineAlign* towards widespread clinical adoption.

**Graphic abstract:**

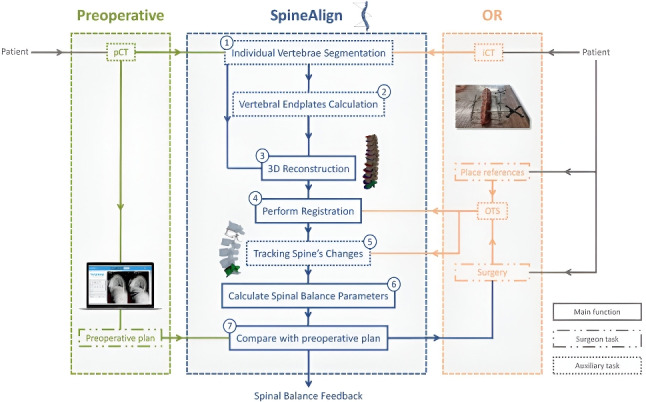

## Introduction

Surgeons currently use commercial software for planning corrections of spinal deformities (SD), which includes tools for assessing preoperative spinal alignment, planning surgery, and predicting surgical outcomes. Computer-assisted spinal surgeries (CASS) leverages preoperative planning, offering advantages such as increased accuracy, reduced invasiveness, enhanced safety, and potentially shorter recovery times, facilitated by technologies like robotic assistance and intraoperative imaging [1].

However, the introduction of these systems has led to a rise in the use of fluoroscopy images and intraoperative computed tomography (CT), resulting in increased radiation exposure. The American National Council on Radiation Protection and Measurements (NCRP) recommends an annual radiation exposure limit of 50 mSv, while the International Commission on Radiological Protection (ICRP) suggests a 5-year average of 20 mSv. Considering the preoperative, intraoperative, and postoperative radiographical studies associated with CASS, patients often approach or exceed these limits [2]. Moreover, despite the benefits of robotic support and intraoperative imaging, intraoperative spinal alignment assessment still relies on visual analysis by the surgeon [2]. Studies indicate that the primary cause of reoperation is incorrect restoration of the patient’s spinal alignment, stemming from inaccurate evaluations made by the surgeon in the operating room (OR) [3].

The increased radiation exposure and the absence of quantitative assessment tools for spinal alignment in the OR pose significant challenges in CASS. There is a pressing need for a radiation-free clinical decision support system (CDSS) capable of providing quantitative feedback on a patient’s spinal alignment. This need is underscored by research indicating that 87% of surgeons require advanced technological tools for assessing spinal alignment during surgery [4].

In this context, the challenge of developing a CDSS to assist surgeons in assessing spinal alignment during SD correction surgery stems from two primary factors currently absent in existing systems. Firstly, there is a lack of mechanisms to continuously monitor changes in the spine’s curvature during surgery. Secondly, the absence of quantitative assessment tools for spinal alignment during surgery presents a significant obstacle [2, 4–6].

Several patents have recently emerged in the field of intraoperative spinal alignment, underscoring both the growing clinical interest and the technical challenges inherent in providing real-time alignment assessment during surgery. These systems aim to enhance surgical precision by incorporating optical tracking, imaging integration, and preoperative planning into the intraoperative workflow. Their existence highlights the unmet need for accurate, actionable feedback during spinal procedures—particularly for deformity correction [7–10].

One notable system for intraoperative spinal alignment is described in Gullotti et al. (2023) [7], which utilizes optical tracking and preoperative planning to assess rod-based spinal correction. This method allows the surgeon to verify alignment by comparing actual implant positions to a pre-defined surgical plan, offering a streamlined workflow for hardware-guided deformity correction. However, this system requires multiple vertebrae to be tracked intraoperatively and relies heavily on implant-specific planning. As a result, its use is generally limited to implant-based procedures, and it lacks the flexibility needed for more general alignment tasks or radiation-free workflows.

Scholl (2024) [8] presents a navigation-integrated solution designed to assist in vertebral alignment by tracking instrumented vertebrae and comparing their intraoperative positions with preoperative alignment targets. It supports continuous monitoring throughout surgical correction and enhances accuracy through real-time visualization. Nevertheless, the approach depends on intraoperative imaging and presumes access to a complete implant plan. This restricts its use to highly structured surgical contexts and complicates adoption in lower-exposure or non-implant-based procedures.

Fujita et al. (2021) [9] introduce a system for spinal alignment monitoring that relies on rigid bodies mounted to the spinous processes of each vertebra and integrates both optical tracking and imaging registration. The system provides quantitative feedback on vertebral motion and enables surgeons to assess intraoperative corrections in real time. Its strength lies in its high-resolution tracking of multiple vertebrae; however, this comes at the cost of significant surgical exposure, requiring each instrumented vertebra to be physically accessible. Additionally, the system assumes deformity correction use cases and lacks an efficient strategy for modeling continuous curvature with minimal tracking input.

Chelala et al. (2022) [10] detail a technique that uses fiducial markers affixed to vertebrae or surgical tools to quantify alignment changes during spinal surgery. Through intraoperative imaging and marker segmentation, it enables measurement of angular deviation or correction post-implantation. While it offers a method for tracking and feedback without relying solely on implants, the approach is still dependent on visible fiducials and intraoperative imaging. It does not provide a real-time curvature model or continuous representation of spinal shape, and its design is best suited for controlled surgical environments with substantial anatomical access.

Most of these patented solutions rely on complex setups involving multiple tracked vertebrae, full anatomical exposure, intraoperative imaging, or implant-specific strategies, limiting their generalization and routine use. This context reinforces the demand for more versatile, minimally invasive solutions that provide real-time curvature feedback without increasing surgical complexity.

This paper introduces *SpineAlign*, a CDSS designed for intraoperative spinal alignment assessment during SD correction, aimed at addressing these existing gaps in the field. Additionally, this work includes a proof of concept (PoC) using a porcine spinal segment to validate all integrated algorithms and functionalities within the system. Thus, this work offers promising solutions to the current challenges encountered in CASS for intraoperative spinal alignment assessment.

### Spinal alignment assessment and treatment

The spine, characterized by gentle curves that evenly distribute the body’s weight and maintain good posture, relies on proper spinal alignment for pain-free movement and support. Unfortunately, SD introduce abnormalities in spinal alignment, impacting up to 60–70% of the aging population [11, 12]. In this section, it is described how spinal alignment is assessed in the case of preoperative SD inspection, including the most commonly used metrics. This clarification serves to enhance the understanding of the developed CDSS.

**Table 1:** Sagittal alignment parameters used to assess spinal alignment

	Identification (units)	Acronym	Description
Cervical params	C2-C7 curvature (°)	C2-C7	Angle between the inferior endplate of C2 and the inferior endplate of C7
	T1 Slope (°)	T1S	Angle between a horizontal line and the superior endplate of T1
	Cervical sagittal vertical axis (mm)	cSVA	Sagittal distance between the vertical line through the center of C2 body (C2 plumbline) and the vertical line through the posterosuperior corner of C7
Thoracic parameters	T1-T12 kyphosis (°)	T1-T12	Angle between the superior endplate of T1 and the inferior endplate of T12
	T4-T12 Kyphosis (°)	T4-T12	Angle between the superior endplate of T4 and the inferior endplate of T12
	T5-T12 Kyphosis (°)	T5-12	Angle between the superior endplate of T5 and the inferior endplate of T12
	Global Thoracic Kyphosis (°)	TKGl	Sum of the arcs of circle that model the thoracic kyphosis as defined by [31] or the angle between the most tilted vertebrae that limit kyphosis
	Thoracolumbar junction angle (°)*	T10-L2	Angle between the superior endplate of T10 and the inferior endplate of L2
Lumbar param	L1-S1 Lordosis (°)	L1-S1	Angle between the superior endplate of L1 and the superior endplate of S1
Spinopelvic params	Pelvic incidence (°)	PI	Angle between the line perpendicular to the sacral plate at its midpoint and the line connecting this point to the femoral head axis
	Pelvic tilt (°)	PT	Angle between a line from the center of the femoral head to the midpoint of the S1 endplate and a vertical reference line drawn through the center of the femoral head
	Sacral slope (°)	SS	Angle between the S1 endplate and the horizontal line
Global params	Sagittal vertical axis (mm)	SVA	The horizontal distance between C7 plumb-line and the posterosuperior corner of S1
	PI-LL Mismatch (°)	PI-LL	Difference between the pelvic incidence and the lumbar lordosis

*Thoracolumbar parameter

Accurate diagnosis and treatment of SD relies on spinal alignment assessment using advanced imaging techniques. Radiographs, offering detailed images of bony structures, are typically the initial modality, aiding in the identification of SD and assessment of spinal alignment. CT proves valuable in specific cases, providing a more intricate view of vertebrae shape and size, essential for pinpointing anatomical abnormalities contributing to deformities. Additionally, CT scans enable 3D reconstruction, aiding the evaluation of vertebral axial rotation [13].

The evaluation of spinal alignment extends to three planes of motion: axial, coronal, and sagittal [14]. The axial plane emerges as a crucial factor in evaluating vertebral rotation, yet the absence of standardized metrics for quantitative measurement remains notable [14]. In contrast, the coronal and sagittal planes dominate the assessment of spinal alignment due to the presence of well-defined metrics facilitating comprehensive evaluation in these dimensions. Coronal alignment involves measuring the horizontal distance between the C7 plumbline and the sacrum’s center, along with the Cobb angle method - Cobb angle is considered the gold standard for quantifying scoliosis [15]. While the Cobb angle simplifies calculations, its 2D nature falls short of describing 3D deformities fully [13].

Sagittal alignment, a complex dimension, exhibits various patterns among asymptomatic individuals. Parameters for assessment span six groups: cervical, thoracic, thoracolumbar, lumbar, spinopelvic, and global (Table 1). Notable metrics include C2-C7 Curvature (C2-C7), T1 Slope (T1S), and Cervical Sagittal Vertical Axis (cSVA) for the cervical spine, T1-T12 Kyphosis (T1-T12) for the thoracic spine, and L1-S1 Lordosis (L1-S1) for the lumbar spine. Spinopelvic parameters, such as Pelvic Incidence (PI), Pelvic Tilt (PT), and Sacral Slope (SS), contribute to evaluating spine-pelvis orientation [16–20]. Global sagittal alignment parameters, including Sagittal Vertical Axis (SVA) and PI-LL Mismatch, measure the relationship and orientation of different spine segments [16–18, 21].

The CDSS developed in this work primarily emphasizes the computation of angular metrics. Thus, metrics such as cSVA and SVA were not included in its development process. Furthermore, spinopelvic parameters were omitted, as they rely on the identification and tracking of femoral heads during surgical procedures. In summary, the CDSS was designed to compute the angulation between all vertebral pairs identified in the CT scan. For instance, if the CT scan includes vertebrae C1, C2, and C3, the system calculates the angles between the endplates of C1-C2, C1-C3, and C2-C3 in both coronal and sagittal planes, providing comprehensive spinal alignment assessment.

## Materials and methods

This work follows a user-centered approach, engaging in discussions with clinical staff specialized in CASS since the beginning to formulate guidelines for intraoperative spinal alignment assessment. Consequently, the development of *SpineAlign* aligns closely with the fundamental requirements of CASS.

A detailed flowchart of the system architecture and user interface components is provided in Appendix 1 (Fig. 9), offering a more comprehensive breakdown of the modules and interactions involved in *SpineAlign*. Three of the four processes that compose *SpineAlign* are well-documented in the literature: individual segmentation of vertebrae [22, 23], calculation of vertebral endplates [24], and intraoperative vertebrae registration [25]. However, in the last process which involves calculating the spinal alignment intraoperatively, we encounter a challenge for which there are no published studies. For this reason, the algorithms developed in the scope of this step are described in this section. Additionally, it discusses how spinal alignment feedback is provided to the surgeon and details the PoC conducted using a porcine spinal segment.

### Spinal alignment calculation

Despite the absence of published studies on intraoperative spinal alignment measurement, recent research on estimating musculoskeletal models from biplane radiographs and CT scans serves as a promising starting point [26, 27]. Fasser et al. created a musculoskeletal model based on biplane radiographs, employing a cubic spline to predict spinal curvature by considering the center of mass (CoM) of the vertebrae [26]. Similarly, Rockenfeller and Müller explored approximating 3D spine curvature using a Bézier curve, offering advantages such as CoM approximation, simulation of spine motion in all anatomical planes, and computational efficiency [27].

This work leverages the inherent advantages of Bézier curves to develop algorithms for intraoperative spinal alignment assessment, modeling the spine as a chain of rigid vertebrae. The mechanical behavior of intervertebral discs is not included, as this simplification enables real-time processing focused on angular alignment rather than full biomechanical modeling. A Bézier curve is defined by a minimum of two points, thus necessitating to track at least two vertebrae by the optical tracking system (OTS) to determine the curve’s start and end points. Furthermore, the algorithms were developed in consideration of additional challenges identified through collaborative meetings with clinical experts, including (i) complexities associated with patient positioning, hindering adherence to anatomical planes; (ii) limitations imposed by minimal invasiveness, impeding individual vertebra tracking; (iii) difficulties in assessing intravertebral movements due to disc plasticity; and (iv) potential alterations to vertebrae shape or spacing resulting from surgical procedures such as osteotomies.

**Fig. 1:**
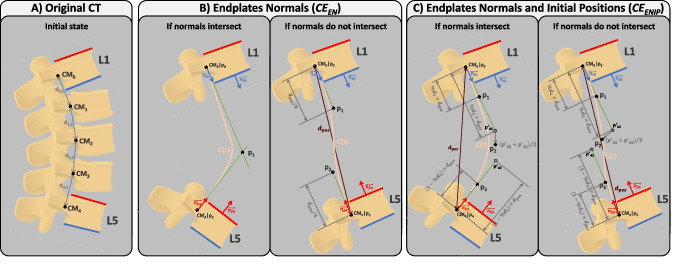
Methods for calculating the control points of the Bézier curve that estimates the curvature of the patient’s spine. Example 1 is used to depict the methods, where L1 and L5 are tracked. The green lines indicate from which CoM the points were estimated, i.e., if the point is connected to $CM_0$ by a green line, it means that it was estimated from $CM_0$. *C*(*t*) is the estimated Bézier curve. Dark red line is the distance between $CM_0$ and $CM_n$ ($d_{pos}$). $\overrightarrow{n_{I0}}$ and $\overrightarrow{n_{S4}}$ are the unit vectors normal to the inferior endplate of L1 and superior endplate of L5, respectively. In dark grey color are indicated distances between points or vectors

**Fig. 2:**
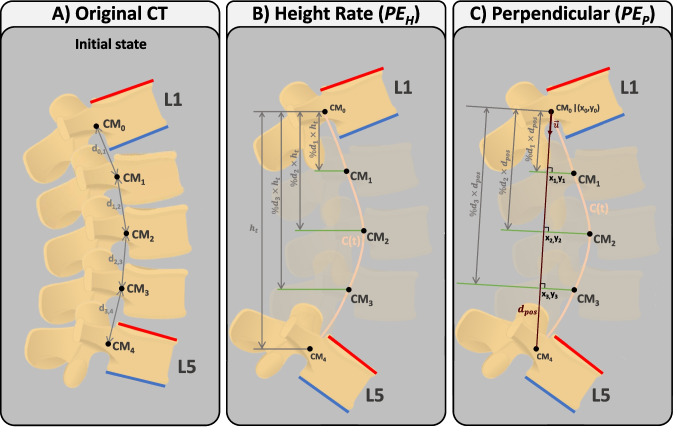
Methods for calculating the position of intermediate vertebrae on the estimated Bézier curve (*C*(*t*)). Example 1 is used to depict the methods, where L1 and L5 are tracked. Green lines represent the lines used to calculate intersection points with *C*(*t*), which are the CoM of intermediate vertebrae. In dark grey color are indicated distances

**Fig. 3:**
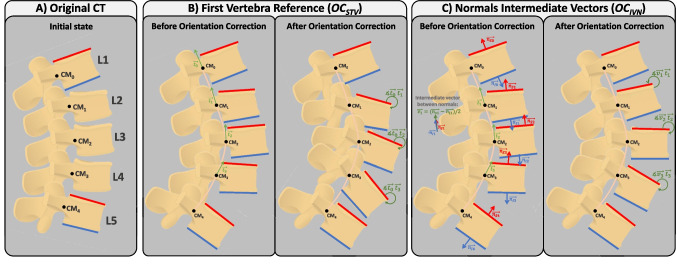
Methods for vertebral orientation correction based on the estimated Bézier curve *C*(*t*). Green right arrows represent tangents of the curve *C*(*t*) at the CoM of the vertebrae. $\overset{\rightarrow }{n}$ represent vectors normal to the endplates of the vertebrae. Arc arrows indicate the direction of the vertebrae according to the algorithm

Hence, the intraoperative spine alignment calculation process involves the exploration of four tasks designed to address the encountered challenges: (i) tracking anatomical planes to calculate parameters in the coronal and sagittal planes (Sect. 2.1.1); (ii) estimating the Bézier curve defining spine curvature, by tracking at least two vertebrae (Sect. 2.1.2, Fig. 1); (iii) determining the position of each untracked vertebra on the Bézier curve (Sect. 2.1.3, Fig. 2); and (iv) adjusting the orientation of each untracked vertebra based on the tangent of the Bézier curve (Sect. 2.1.4, Fig. 3). These methods are described in the following sections.

Appendix 2 presents the nomenclature and definition of the parameters used to describe the developed algorithms. Throughout the text, Example 1 illustrates a scenario where L1 and L5 are tracked and serve as a reference for explaining the algorithms in the text. In Figs. 1, 2, and 3 are depicted the methods developed in the scope of intraoperative spinal alignment assessment, resorting to Example 1.

#### Anatomical planes tracking

In the operating room (OR), patients undergo dynamic movements on the operating table based on the requirements of the surgical procedures. In the 3D window of *SpineAlign*, the vertebrae move freely to accommodate the patient’s positional changes. However, on the axial, coronal, and sagittal views, adherence to anatomical planes is crucial because the patient’s spinal alignment parameters depend on the angles between the vertebral endplates in the sagittal and coronal planes. *SpineAlign* addresses this by designating the superior tracked vertebra ($VR_0$) as a fixed reference during surgery. Following vertebrae registration, to ensure $VR_0$ remains the reference, the software calculates transformation matrices for other vertebrae relative to this one (${}_{{VR_i}}^{\hspace{2.0pt}{VR_0}}T$). This approach maintains the accuracy of anatomical plane tracking and facilitates precise spinal alignment calculation.

Given the transformation matrix of each reference sent by the OTS as ${}_{\hspace{2.0pt}{VR_i}}^{{OTS}}T$, the transformation matrix of the $i^{th}$ tracked vertebra ($VR_i$) relative to $VR_0$ is calculated as follows:

1 $$\begin{aligned} {}_{{VR_i}}^{\hspace{2.0pt}{VR_0}}T\hspace{2.0pt}= \hspace{2.0pt}{}_{\hspace{4.0pt}{VR_0}}^{{OTS}}T^{-1}\hspace{4.0pt}{}_{\hspace{2.0pt}{VR_i}}^{{OTS}}T\quad i=1,\ldots ,n-1 \end{aligned}$$

where *n* is the number of tracked vertebrae. In Example 1 (L1 and L5 tracked), L1 is used as reference ($VR_0$) and the position of L5 ($VR_1$) is calculated relative to $VR_0$ following Eq. 1: ${}_{{VR_1}}^{\hspace{2.0pt}{VR_0}}T$.

#### Spinal curve estimation

During surgery, the absence of information regarding the untracked vertebrae requires the use of Bézier curves for spinal curvature estimation. A Bézier curve *C*(*t*) is dependent on control points to shape its curvature, beginning at $p_0$ and ending at $p_{k-1}$, where k represents the number of control points [28]. To estimate spinal curvature, control points are derived from the positions of tracked vertebrae, with $p_0$ denoting the position of the CoM of the superior tracked vertebra ($CM_0$), and $p_{k-1}$ representing the CoM position of the inferior tracked vertebra ($CM_{n-1}$, where *n* is the number of vertebrae in the segment). Estimating the control points corresponds to estimating the Bézier curve they define. Consequently, two approaches are employed for estimating the Bézier curve of a segment: (i) based on the endplate normals of tracked vertebrae ($CE_{EN}$) and (ii) incorporating endplate normals and initial positions of the vertebrae in the CT scan ($CE_{ENIP}$). The endplate’s normals are imaginary lines or vectors perpendicular to the surface of the vertebral endplates in coronal and sagittal planes.

**Algorithm 1:**
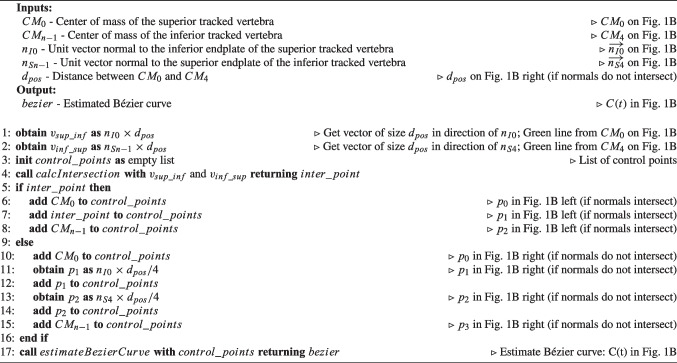
Pseudocode for Curve Estimation based on Endplates’ Normals of tracked vertebrae ($CE_{EN}$). Comments make the connection to Fig. 1B.

**Algorithm 2:**
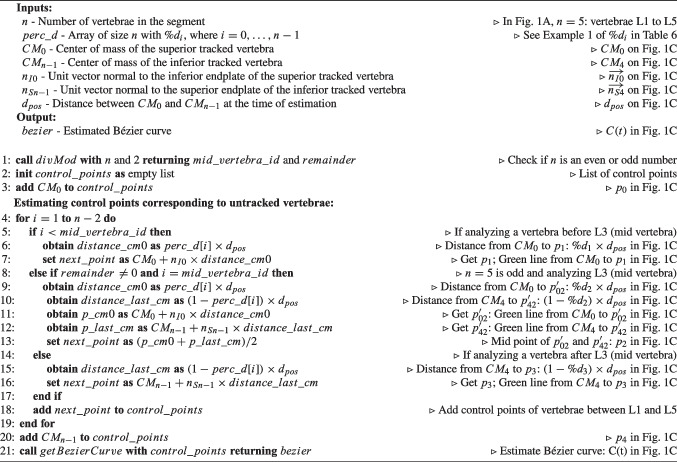
Pseudocode for Curve Estimation based on Endplates’ Normals of tracked vertebrae and Initial Position of the vertebrae on CT scan ($CE_{ENIP}$). Comments make the connection to Fig. 1C.

Algorithm 1 contains the pseudocode corresponding to $CE_{EN}$, which is complemented by a visual representation of Example 1 (L1 and L5 tracked) in Fig. 1B for better understanding. In $CE_{EN}$, the simplicity of the method becomes apparent when assessing the normals of the tracked vertebrae. In Example 1, if the normals of the inferior endplate of L1 ($\overrightarrow{n_{I0}}$) and superior endplate of L5 ($\overrightarrow{n_{S4}}$) intersect (Fig. 1B left), three control points are used: (i) $CM_0$, which is the CoM of L1 ($p_0$); (ii) the intersection of the two normals ($p_1$); and (iii) $CM_4$, which is the CoM of L5 ($p_2$). In the case that normals do not intersect (Fig. 1B right), the distance between $CM_0$ and $CM_4$ at estimation time ($d_{pos}$ in Fig. 1B right) is employed. Four control points are then used, incorporating: (i) $CM_0$ ($p_0$); (ii) a point at $d_{pos}/4$ distance from $CM_0$ along the normal of L1 ($p_1$); (iii) a point at $d_{pos}/4$ distance from $CM_4$ along the normal of L5 ($p_2$); and (iv) $CM_4$ ($p_3$). Using $d_{pos}/4$ allows the four points that control the curve to be equally spaced.

Algorithm 2 contains the pseudocode corresponding to $CE_{ENIP}$, which is complemented by a visual representation of Example 1 (L1 and L5 tracked) in Fig. 1C for better understanding. In $CE_{ENIP}$, the method involves calculating distances between the CoM of consecutive vertebrae in the initial CT scan to calculate one control point for each vertebra. These distances, expressed as a percentage of the total distance from $CM_0$ to $CM_n$ ($\%d_i$ in Table 6), are then used to calculate the control points for estimating the new curvature of the spine. One control point of the curve is calculated for each vertebra, with the CoM of the superior and inferior tracked vertebrae serving as the starting and ending points ($p_0$ and $p_{n-1}$), respectively. The control points of the intermediate vertebrae are calculated from $CM_0$ or $CM_{n-1}$, depending on whether the vertebra is closer to $CM_0$ or $CM_{n-1}$ in terms of number of vertebrae. Considering Example 1, L2 ($CM_1$) is closer to L1 ($CM_0$) than L5, so control point $p_1$ is calculated from $CM_0$ ($p_1=CM_0+n_{I0}\times \%d_1\times d_{pos}$), as shown by the green line connecting $CM_0$ and $p_1$ in Fig. 1C. On the other hand, L4 ($CM_3$) is closer to L5 ($CM_4$) than L1, so control point $p_3$ is calculated from $CM_4$ ($p_3=CM_4+n_{S4}\times (1-\%d_3)\times d_{pos}$), as shown by the green line connecting $CM_4$ and $p_3$ in Fig. 1C. However, L3 ($CM_2$) is equally distant from L1 ($CM_0$) and L5 ($CM_4$). Whenever there is an odd number of intermediate vertebrae, the control point associated with the mid vertebra will be the midpoint of the points calculated from $CM_0$ and $CM_{n-1}$, thus $CM_2$ in Example 1 (CoM of L3) will be the midpoint between the points calculated from $CM_0$ and from $CM_4$ ($p'_{02}$ and $p'_{42}$ in Fig. 1C, respectively):

$$ CM_2 = \frac{p'_{02}+p'_{42}}{2} = \frac{[CM_0+n_{I0}\times \%d_2\times d_{pos}] + [CM_4+n_{S4}\times (1-\%d_2)\times d_{pos}]}{2} $$

Both methods for estimating spinal curvature are described using the sagittal plane as an example, but this implementation has also been used to estimate spinal curvature in the coronal plane.

#### Vertebrae position estimation

In the process of estimating spinal curvature and achieving intraoperative spinal alignment assessment, the challenge lies in accurately determining the position of intermediate vertebrae located between two tracked vertebrae. Two methods have been developed based on the distance between the vertebrae in the initial CT scan: (i) resorting to the height difference between the two tracked vertebrae ($PE_{H}$) and (ii) resorting to the perpendiculars of the straight line connecting the CoM of the two tracked vertebrae ($PE_{P}$).

**Algorithm 3:**
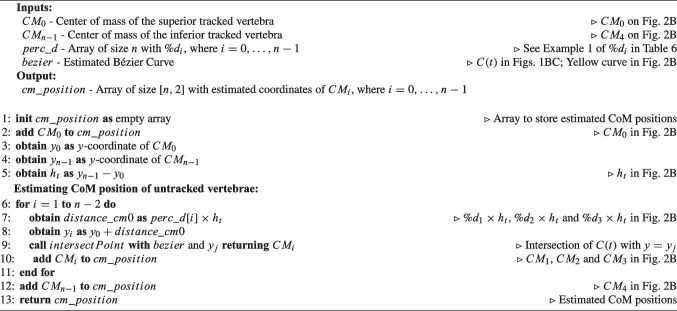
Pseudocode for Position Estimation based on Height’s difference between the two tracked vertebrae ($PE_{H}$). Comments make the connection to Fig. 2B.

Algorithm 3 contains the pseudocode corresponding to $PE_H$, which is complemented by a visual representation of Example 1 (L1 and L5 tracked) in Fig. 2B for better understanding. In Example 1, $h_t$ is the height between the CoM of L1 and L5 ($CM_0$ and $CM_4$ in Fig. 2B), which are being tracked. Then, the *y*-coordinate of $CM_1$, $CM_2$, and $CM_3$ ($y_1$, $y_2$, and $y_3$) are calculated from *y*-coordinate of $CM_0$ ($y_0$): $y_1=y_0+\%d_1\times h_t$; $y_2=y_0+\%d_2\times h_t$; and $y_3=y_0+\%d_3\times h_t$. The points on the Bézier curve (*C*(*t*)) where $y=y_1$, $y=y_2$, and $y=y_3$ correspond to the CoM of L1, L2, and L3 ($CM_1$, $CM_2$, and $CM_3$ in Fig. 2B).

**Algorithm 4:**
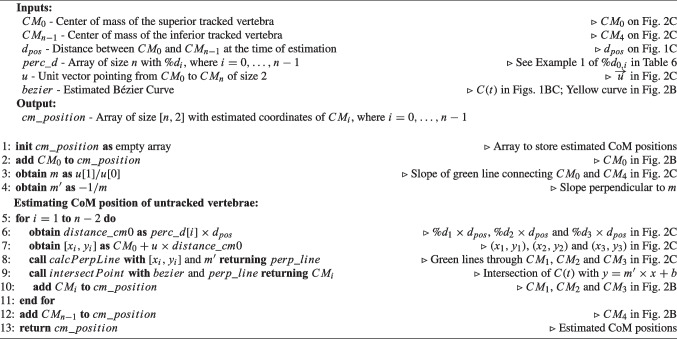
Pseudocode for Position Estimation based on the Perpendiculars of the straight line connecting the CoM of the two tracked vertebrae ($PE_{P}$). Comments make the connection to Fig. 2C.

Algorithm 4 contains the pseudocode corresponding to $PE_P$, which is complemented by a visual representation of Example 1 in Fig. 2C for better understanding. In Example 1, $\overrightarrow{u}$ in Fig. 2C is the unit vector pointing from the CoM of L1 ($CM_0$) to the CoM of L5 ($CM_4$). *m* is the slope of the line connecting $CM_0$ and $CM_4$ (dark red line in Fig. 2C), obtained as $m=u_y/u_x$. The negative reciprocal of *m* is the slope of any line perpendicular to the line connecting $CM_0$ and $CM_4$: $m_p=-1/m$. We want to estimate the position of L1, L2, and L3 ($CM_1$, $CM_2$, and $CM_3$) using three perpendicular lines (green lines in Fig. 2C) on the form:

$$ y_i=m_p x_i+b_i,\quad i=1,2,3 $$

In order to find $b_i$, we need to know a point $(x_i,y_i)$ that belongs to the perpendicular lines. We know that $(x_i,y_i)$ will belong to the line connecting $CM_0$ and $CM_4$ because the perpendicular will intersect it at any point. Thus, we can use $\overrightarrow{u}$ as the direction to follow from $CM_0$ to get each point $(x_i,y_i)$. The distance from $CM_0$ to the point $(x_i,y_i)$ is given by $\%d_i\times d_{pos}$, thus we get the point through $(x_i,y_i)=CM_0+\overrightarrow{u}\times \%d_i\times d_{pos}$, obtaining the points $(x_1,y_1)$, $(x_2,y_2)$, and $(x_3,y_3)$ in Fig. 2C. Finally, three line equations are found:

$$ {\left\{ \begin{array}{ll} y_1=m_p x_1+b_1,\quad \text {corresponding to }CM_1 \text { (L2)}\\ y_2=m_p x_2+b_2,\quad \text {corresponding to }CM_2 \text { (L3)}\\ y_3=m_p x_3+b_3,\quad \text {corresponding to }CM_3 \text { (L4)} \end{array}\right. } $$

The intersection of each of these lines with the estimated Bézier curve (*C*(*t*)) corresponds to the estimation position of the CoM of the untracked vertebrae: $CM_1$, $CM_2$, and $CM_3$.

Both methods for estimating vertebrae’s position are described using the sagittal plane as an example, but this implementation has also been used to estimate vertebrae’s position in the coronal plane.

#### Vertebrae orientation correction

This step focuses on aligning the intermediate vertebrae with the estimated Bézier curve (*C*(*t*)). The derivable nature of the Bézier curve equation enables the calculation of the tangent vector at any point along the curve by using its first derivative $C'(t)$. The tangent represents the slope and direction of the curve at that specific point. To correct the orientation of intermediate vertebrae, two methods were developed that use the tangent information: (i) orienting to the superior tracked vertebra ($OC_{STV}$) and (ii) considering the intermediate vector to the superior and inferior endplates’ normals of tracked vertebrae ($OC_{IVN}$).

Algorithm 5 contains the pseudocode corresponding to $OC_{STV}$, which is complemented by a visual representation of Example 1 in Fig. 3B for better understanding. In $OC_{STV}$, the superior tracked vertebra is used as a reference for orientation. The orientation of intermediate vertebrae is corrected by aligning the tangents in the CoM of these vertebrae ($t_i$) with the tangent of the Bézier curve at the CoM of the superior tracked vertebra ($t_0$). The formula used for this calculation is:

2 $$\begin{aligned} \measuredangle \overrightarrow{t_0}\hspace{2.0pt}\overrightarrow{t_i} = \arccos {\left( \frac{\overrightarrow{t_0}\cdot \overrightarrow{t_i}}{|\overrightarrow{t_0}||\overrightarrow{t_i}|}\right) }, \quad i=1,\ldots ,n-2 \end{aligned}$$

where $\overrightarrow{t_0}\cdot \overrightarrow{t_i}$ is the dot product between $\overrightarrow{t_0}$ and $\overrightarrow{t_i}$. In Example 1, it is necessary to align L2, L3, and L4 with L1 ($CM_0$). $C'(t)$ is evaluated at $CM_1$, $CM_2$, and $CM_3$, returning vectors tangent to the curve at these points: $\overrightarrow{t_1}$, $\overrightarrow{t_2}$, and $\overrightarrow{t_3}$ in Fig. 3B. The rotation employed to L2, L3, and L4 are obtained as $\measuredangle \overrightarrow{t_0}\hspace{2.0pt}\overrightarrow{t_1}$, $\measuredangle \overrightarrow{t_0}\hspace{2.0pt}\overrightarrow{t_2}$, and $\measuredangle \overrightarrow{t_0}\hspace{2.0pt}\overrightarrow{t_3}$, respectively, following Eq. 2.

**Algorithm 5:**
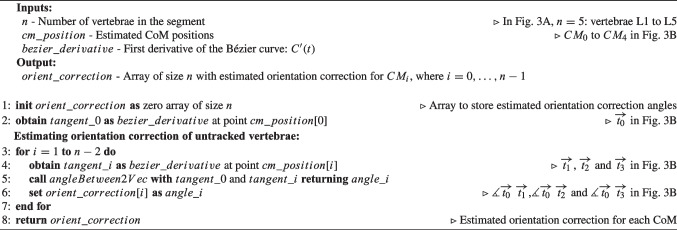
Pseudocode for Orientation Correction based on the Superior Tracked Vertebra ($CO_{STV}$). Comments make the connection to Fig. 3B.

**Algorithm 6:**
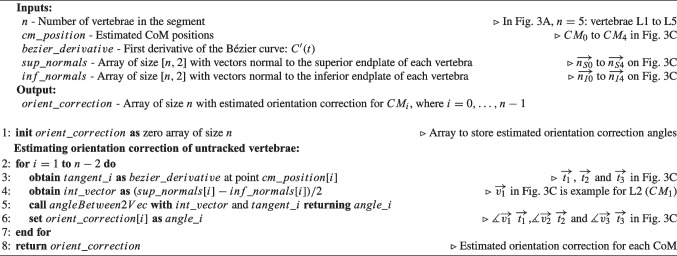
Pseudocode for Orientation Correction considering the Intermediate Vector to the superior and inferior endplates’ Normals of tracked vertebrae ($CO_{IVN}$). Comments make the connection to Fig. 3C.

Algorithm 6 contains the pseudocode corresponding to $OC_{IVN}$, which is complemented by a visual representation of Example 1 in Fig. 3C for better understanding. In this method, the orientation correction is achieved by aligning the intermediate vector of the superior and inferior endplates ($\overrightarrow{v_i}$) with the tangent vector to the Bézier curve at the CoM of the respective vertebra ($\overrightarrow{t_i}$). The intermediate vector $\overrightarrow{v_i}$ for each intermediate vertebra ($CM_i$) is determined by subtracting the normal vector to the inferior endplate ($\overrightarrow{n_{Ii}}$) from the normal vector to the superior endplate ($\overrightarrow{n_{Si}}$) and dividing by 2: $\overrightarrow{v_i}=\frac{\overrightarrow{n_{Ii}}-\overrightarrow{n_{Si}}}{2}$. The orientation correction angle is then calculated as:

3 $$\begin{aligned} \measuredangle \overrightarrow{v_i}\hspace{2.0pt}\overrightarrow{t_i} = \arccos {\left( \frac{\overrightarrow{v_i}\cdot \overrightarrow{t_i}}{|\overrightarrow{v_i}||\overrightarrow{t_i}|}\right) }, \quad i=1,\ldots ,n-2 \end{aligned}$$

where $\overrightarrow{v_i}\cdot \overrightarrow{t_i}$ is the dot product between $\overrightarrow{t_0}$ and $\overrightarrow{t_i}$. As for Example 1, $C'(t)$ is evaluated at $CM_1$, $CM_2$, and $CM_3$, returning vectors tangent to the curve at these points: $\overrightarrow{t_1}$, $\overrightarrow{t_2}$, and $\overrightarrow{t_3}$ in Fig. 3C. The intermediate vectors of the endplates’ normals of L1, L2, and L3 are calculated as: $\overrightarrow{v_1}=\frac{\overrightarrow{n_{I1}}-\overrightarrow{n_{S1}}}{2}$; $\overrightarrow{v_2}=\frac{\overrightarrow{n_{I2}}-\overrightarrow{n_{S2}}}{2}$, and $\overrightarrow{v_3}=\frac{\overrightarrow{n_{I3}}-\overrightarrow{n_{S3}}}{2}$. $\overrightarrow{v_1}$ is depicted in Fig. 3C. Lastly, L1, L2, and L3 orientation is corrected by rotating each of them by the angles $\measuredangle \overrightarrow{v_1}\hspace{2.0pt}\overrightarrow{t_1}$, $\measuredangle \overrightarrow{v_2}\hspace{2.0pt}\overrightarrow{t_2}$, and $\measuredangle \overrightarrow{v_3}\hspace{2.0pt}\overrightarrow{t_3}$, respectively, following Eq. 3, ensuring proper alignment of the intermediate vertebrae in the direction of their endplates.

**Fig. 4:**
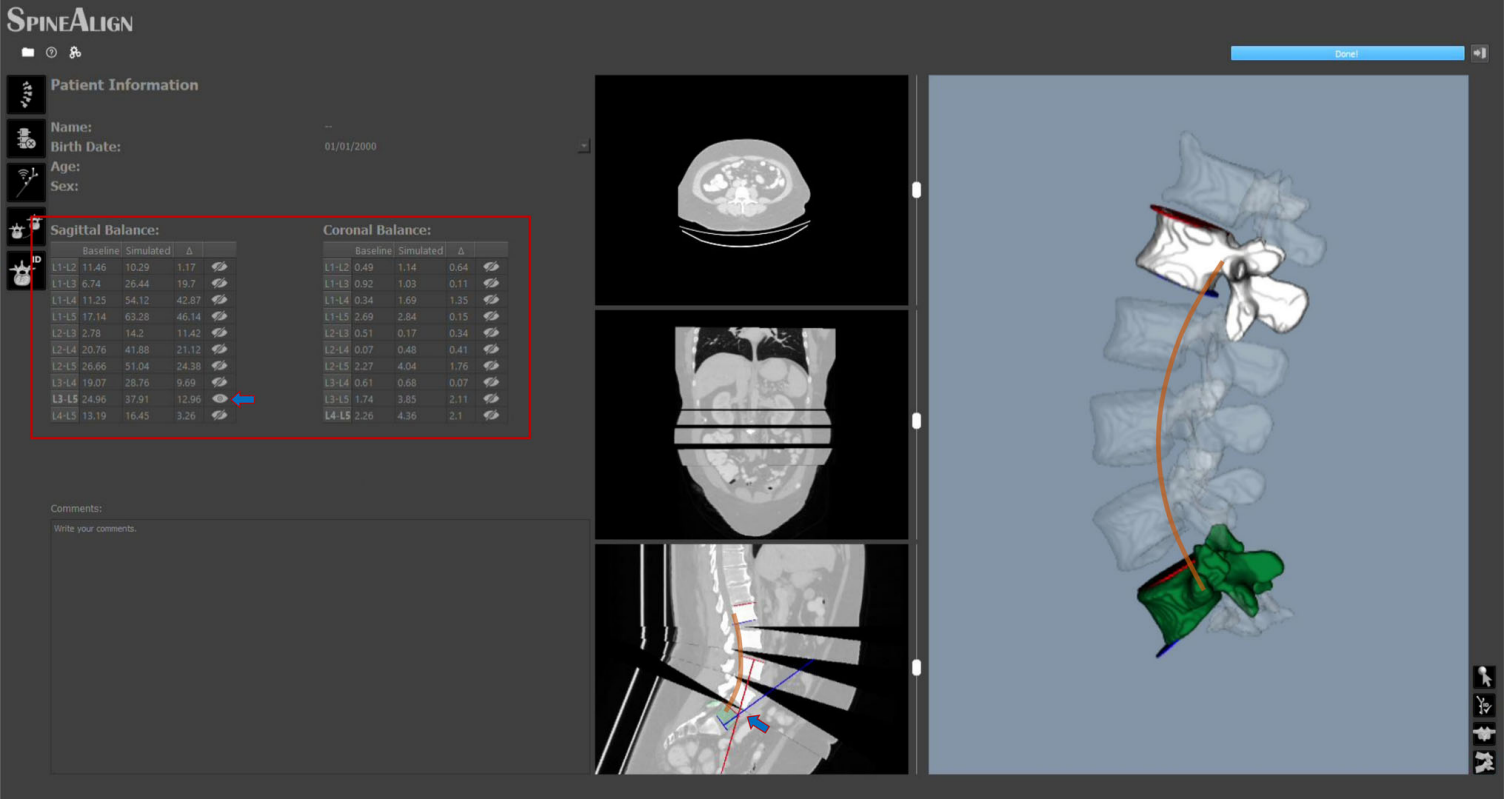
*SpineAlign* GUI showing the feedback on the patient’s spinal alignment provided to the surgeon during surgery. The red box indicates the tables showing the alignment parameters at the beginning of surgery (“Baseline”), during surgery (“Simulated”), and the correction amplitude (“$\Delta$”). The arrows indicate the toggle of the visualization of the endplate normals in the sagittal plane. The orange curves are placed to illustrate the estimated Bézier curve and are not part of the feedback provided

Similarly to the methods for calculating the control points of the Bézier curve ($CE_{EN}$ and $CE_{ENIP}$) and the position of the intermediate vertebrae ($PE_H$ and $PE_P$), the orientation correction ($OC_{STV}$ and $OC_{IVN}$) is estimated in both sagittal and coronal planes. While in the other methods an independent analysis of each plane can be performed, in this case of orientation correction, the corrections calculated in each plane for each vertebra must be combined. To describe the combination of the rotations on both planes, let us define the rotation angle for the vertebra with CoM $CM_i$ on the sagittal plane as $\theta _{si}$ and on the coronal plane as $\theta _{ci}$. Considering the left, anterior, and inferior (LAI) scan orientation, the rotation vector of the correction needed for the vertebra will be $r_\theta =[\theta _{sj},\theta _{cj},0]$. Both rotations are combined so that the correction is made in 3D space. To do this, the quaternion representing the application of both rotations was computed [29]. Afterwards, the corresponding transformation matrix can be calculated and applied to the vertebra in question.

### Spinal alignment feedback

The graphical user interface (GUI) of *SpineAlign* serves as a comprehensive tool, offering quantitative feedback to the surgeon regarding the patient’s spinal alignment. This interface, depicted in Fig. 4, dynamically updates the position and orientation of vertebrae in both 3D and multiplanar views. Calculation of angles between endplates for all vertebral pairs is a key feature, providing detailed information on spinal alignment parameters. The angles are consistently computed using the superior endplate of the superior vertebra and the inferior endplate of the inferior vertebra, aligning with established literature practices. The GUI includes two tables displaying the angle between any two vertebral endplates of different vertebrae in the segment evaluated in coronal and sagittal planes (red box in Fig. 4). Both tables consist of a “Baseline” column representing values after initial registration, a “Simulated” column reflecting real-time changes during surgery, and a “$\Delta$” column highlighting the difference between baseline and simulated values, offering quantitative insights into spinal alignment and the extent of corrections made. Additionally, the GUI enables the visualization of endplates’ normals for each vertebral pair on sagittal and coronal views, enhancing the surgeon’s ability to visually assess spinal alignment (arrows in Fig. 4).

**Fig. 5:**
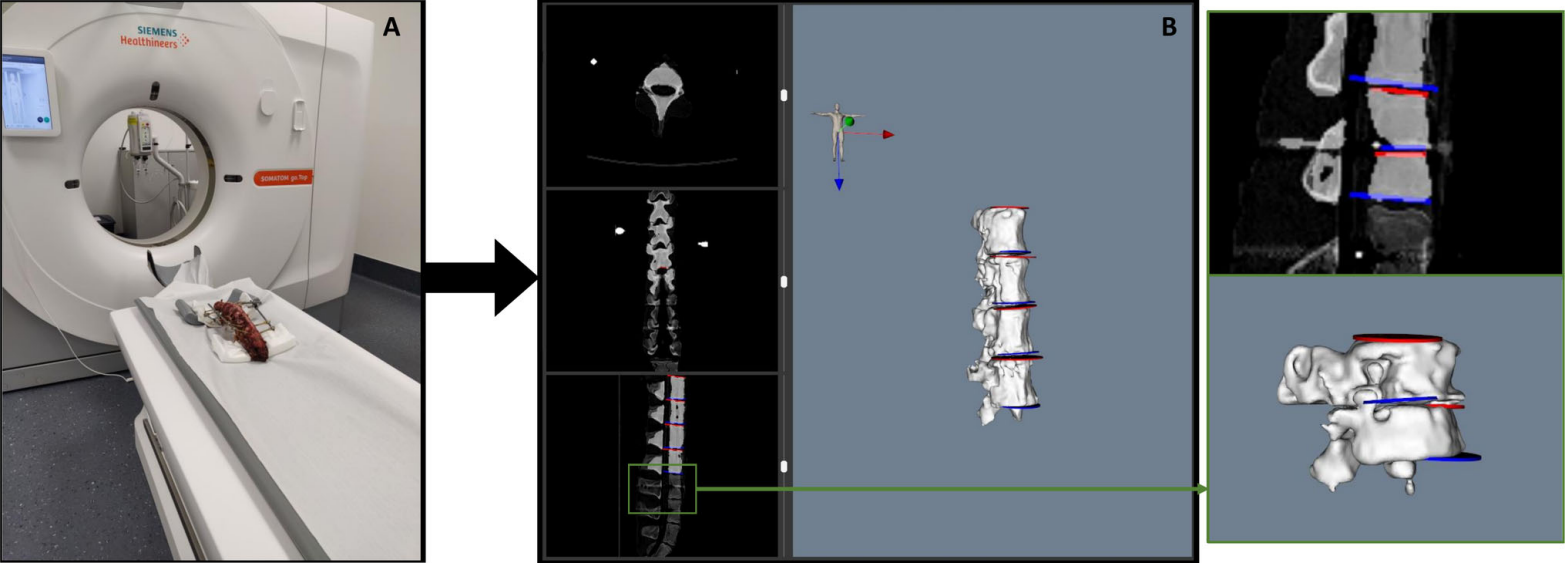
A) CT scan of pig’s spine and B) respective segmentation and endplates calculation. Right side highlights the segmentation of one of the vertebra used to attach the screws of the fixation system, showing that the vertebra was divided in two parts

### Proof of concept

A PoC was conducted using a segment of a porcine spine with 8 vertebrae (T13 to L6). The goal of the proof of concept is to investigate the accuracy of the methods described before in calculating the alignment of the spine of the untracked vertebrae at the beginning and end of surgery. The mobility of the pig spine is different from that of the human spine, which may cause the shape of the spine at the end of surgery to be unrealistic, i.e., the final shape of the pig spine may not occur in a hospital setting when it comes to correcting SDs in humans. However, this does not affect the ability to draw conclusions about the accuracy of the system; in fact, it may even indicate that the system is capable of assessing the alignment of the patient’s spine when its shape is unusual. With this in mind, the protocol defined in the following paragraphs was implemented to transition from a neutral lumbar segment to a lordotic segment of the porcine spine.

Challenges associated with the experiment included addressing intravertebral motion between the surgical procedure and taking the CT scan, necessitating the development of a fixation system to maintain spine curvature stability. The fixation system was attached to the vertebral bodies of the top 2 (T13 and T14) and bottom 2 vertebrae (L5 and L6) using screws. After a surgeon with 13 years of experience place the fixation system to ensure that the spinal curvature does not change, a high-resolution CT scan was performed, which is defined as the preoperative CT (Fig. 5A). By segmenting the vertebrae and calculating the endplates, the 4 vertebrae used to attach the fixation system were excluded from the PoC because the screws divided the vertebrae in two segments (right side of Fig. 5), leaving 4 intermediate vertebrae (L1 to L4) as the focus of the experiment.

A Ponte osteotomy protocol was developed, aiming to obtain angles between vertebrae pairs at the procedure’s start and end (“Baseline” and “Simulated” columns in tables of *SpineAlign* GUI, respectively). To simulate the correction of a spinal deformity, a Ponte osteotomy at L3 was performed, which consists of a vertebral resection to increase the lordosis of the porcine spine. The surgical procedure was performed by a surgeon with 13 years of experience in spinal surgery. No indication was given as to the correction to be made, since the objective is to determine which of the methods developed best approximates the real curvature of the spine, and therefore, the correction made by the surgeon may be of any magnitude.

References were securely attached to L1 and L4 spinal processes by mean of a screw. Both vertebrae were then registered using point-based and surface registration steps [25]. After performing osteotomy, the fixation system is unlocked. A new curvature is given to the porcine spine, and the fixation system is locked again, ensuring that the spine curvature does not change until a new high-resolution CT scan is performed. This CT scan, performed after the surgical procedure, is defined as postoperative CT.

**Fig. 6:**
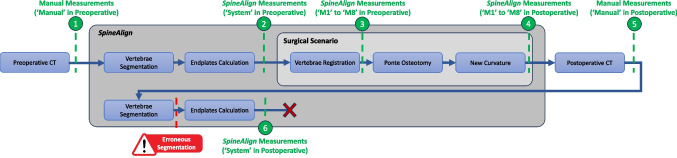
Flowchart of the proof of concept (PoC) performed using a porcine spine segment. In green are identified the measurements performed throughout the PoC. Inside brackets is made the connection with Table 3. The segmentation performed by *SpineAlign* in the postoperative CT scan was not correct, which is identified as “Erroneous Segmentation.” The red “X” at the bottom indicates that no more steps were performed

**Table 2:** Methods for quantitative spinal alignment assessment, as a result of the combination of the methods implemented for curve estimation, position estimation, and orientation correction

	Curve Estimation		Position Estimation		Orientation Correction	
Method	$CE_{EN}$	$CE_{ENIP}$	$PE_{H}$	$PE_{P}$	$O_{STV}$	$O_{IVN}$
M1	X		X		X	
M2		X		X	X	
M3	X			X	X	
M4		X	X		X	
M5	X		X			X
M6		X		X		X
M7	X			X		X
M8		X	X			X

Figure 6 shows the flowchart of the PoC, beginning with the preoperative CT scan and ending with the proposed spinal alignment assessment made on the postoperative CT scan. Figure 6 shows the measurements made throughout the PoC, making the connection with Table 3, where the results of each assessment are reported. Numbers 1 and 6 in Fig. 6 correspond to the preoperative and postoperative manual assessments, respectively. The manual measurements were performed by a surgeon with 24 years of experience who was not present during the surgical procedure to ensure blinded assessments. These measurements were made with commercially available software used in the planning of spinal deformity correction surgeries and are the gold standard used for comparison with the system measurements.

The complexity of the intraoperative spinal alignment calculation, involving the algorithms developed for spinal curve estimation, vertebrae position estimation, and orientation correction, led to the development of eight distinct evaluation methods (M1 to M8), each addressing specific aspects of the process. These methods, detailed in Table 2, provided a comprehensive approach to assessing the effectiveness of the *SpineAlign* system in a simulated surgical scenario.

## Results

This section presents the results obtained in the PoC and is divided into two sections: (i) clinical outcomes and (ii) *SpineAlign* accuracy. The first section consists in the presentation of the main clinical insights regarding the results obtained through the PoC of a surgery for SD correction, comparing the porcine spine before and after the procedure. The second section aims to evaluate the overall accuracy of *SpineAlign* and, at the same time, conclude which methods (M1 to M8 in Table 2) are more suitable for this application.

**Fig. 7:**
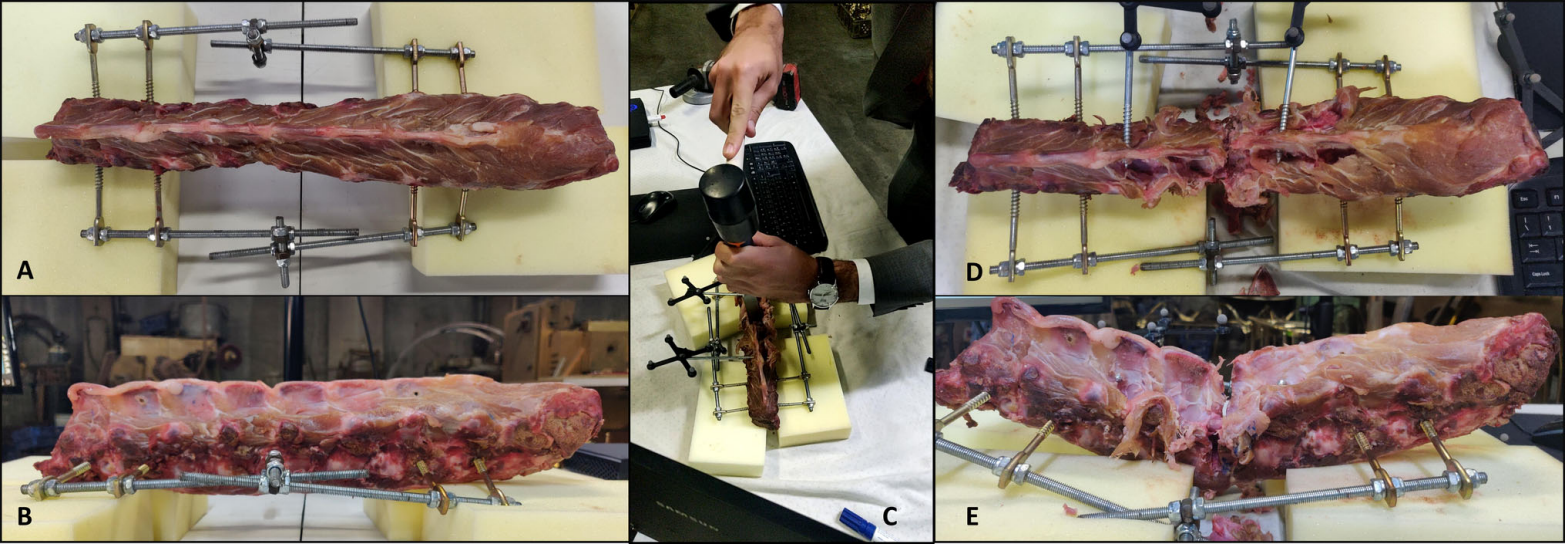
Porcine spine in different stages of the proof of concept. **A** posterior view of the porcine spine before the surgical procedure, showing coronal alignment; **B** left view of the porcine spine before the surgical procedure, showing sagittal alignment; **C** Ponte osteotomy being performed at L3 with references placed at L1 and L4; **D** posterior view of the porcine spine at the end of surgical procedure, showing coronal alignment; **E** left view of the porcine spine at the end of surgical procedure, showing sagittal alignment

It is important to note the time required to assemble the setup and register the vertebrae. In the proof of concept, it took about 12 min to assemble the setup, while the process of registering the vertebrae took 16 min (including placing the references on the vertebrae). Therefore, using the system will require approximately 28 min to complete these tasks.

### Clinical outcomes

In terms of coronal alignment, the porcine spine did not show any scoliosis preoperatively, as shown in Fig. 7A. Sagittally, although we tried, it was not possible to fixate the spine with any degree of lordosis because the porcine spine naturally does not allow this behavior. Therefore, the porcine spine was preoperatively immobilized with no lordosis or kyphosis, as shown in Fig. 7B.

By resecting part of L3 (Fig. 7C), it was possible to give a new curvature to the porcine spine. In the coronal plane, the alignment of the spine continued without showing any degree of scoliosis, as shown in Fig. 7D. On the other hand, in the sagittal plane, a “V” shape of the spine was obtained with the apex in the area of the vertebral resection, as shown in Fig. 7E.

**Table 3:** Comparison of manually assessing the spinal alignment (Manual) and using *SpineAlign*, including the root mean square error (RMSE) associated to each measurement

	Coronal (°)							Sagittal (°)						
	L1-L2	L1-L3	L1-L4	L2-L3	L2-L4	L3-L4	RMSE	L1-L2	L1-L3	L1-L4	L2-L3	L2-L4	L3-L4	RMSE
Preoperative														
Manual (1)	1.44	2.90	1.19	3.80	2.09	3.93	0.00	1.98	4.59	1.21	6.29	1.25	1.08	0.00
System (2)	1.75	2.34	0.45	2.68	0.11	5.71	1.25	1.86	5.37	0.90	5.34	0.93	1.87	0.63
M1 (3)	2.18	3.44	0.47	3.35	0.38	5.86	1.17	9.46	12.65	0.95	5.03	8.57	9.18	6.35
M2 (3)	1.97	2.99	0.51	3.11	0.32	5.68	1.11	10.75	14.07	0.94	5.15	9.45	10.19	7.28
M3 (3)	2.21	3.79	0.45	3.68	0.34	6.22	1.31	13.56	12.66	1.07	4.95	12.8	9.31	8.19
M4 (3)	1.99	3.04	0.48	3.05	0.31	5.56	1.10	10.86	14.22	0.96	5.24	10.24	9.40	7.33
M5 (3)	1.58	2.80	0.50	2.69	1.44	4.79	0.71	4.24	7.89	0.97	5.54	4.61	3.02	2.30
M6 (3)	1.73	2.47	0.49	2.53	1.52	5.03	0.81	3.97	7.42	1.04	4.81	3.52	3.47	2.04
M7 (3)	1.60	2.72	0.47	2.74	1.43	4.82	**0**.**70**	4.86	8.34	1.01	5.73	4.24	3.65	2.53
M8 (3)	1.76	2.44	0.46	2.65	1.55	4.89	0.75	3.59	7.25	1.03	4.64	3.44	3.39	**1**.**94**
Postoperative														
Manual (5)	0.83	3.43	0.17	2.60	1.01	0.17	0.00	4.00	17.25	19.66	17.60	20.00	2.41	0.00
System* (6)	24.55	22.92	21.25	10.71	9.04	45.35	24.35	25.80	15.61	27.37	2.19	13.94	12.20	12.30
M1 (4)	0.24	0.79	0.03	3.21	2.40	4.02	2.02	9.27	12.40	19.93	1.29	8.81	3.13	8.59
M2 (4)	0.69	0.98	0.06	3.85	2.81	4.27	2.15	8.98	13.34	19.88	2.51	9.05	2.14	8.04
M3 (4)	0.23	0.79	0.05	3.21	2.38	4.04	2.02	9.57	12.54	19.90	1.12	8.48	2.97	8.74
M4 (4)	0.68	0.98	0.05	3.84	2.81	4.26	2.14	8.58	13.20	19.89	2.77	9.46	2.29	7.84
M5 (4)	1.19	3.38	0.05	0.04	3.29	0.21	**1**.**41**	5.76	11.13	19.89	3.51	12.27	4.41	7.10
M6 (4)	1.62	3.19	0.06	0.58	3.71	0.01	1.42	5.52	12.14	19.91	4.76	12.53	3.43	6.46
M7 (4)	1.17	3.36	0.04	0.04	3.29	0.20	**1**.**41**	6.04	11.27	19.90	3.37	12.00	4.28	7.19
M8 (4)	1.63	3.19	0.04	0.58	3.73	0.01	1.43	5.07	11.94	19.91	5.01	12.98	3.61	**6**.**31**

The number inside brackets indicates the measurement number in Fig. 6. M1 to M8 refers to the methods developed in the scope of intraoperative spinal alignment assessment, as detailed in Table 2

*Results based on erroneous segmentation

The bold entries indicate the best-performing result across the compared methods for each plane (sagittal and coronal). They highlight the lowest values, demonstrating which method achieved superior performance in each plane

**Fig. 8:**
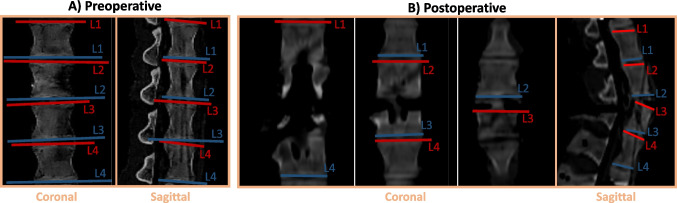
Manual spinal alignment assessment on preoperative (**A**) and postoperative (**B**) CT scans. Superior endplates are shown in red color, while inferior endplates are shown in blue. Vertebra identification allows to make the correspondence between the endplate and the vertebra it belongs to

### *SpineAlign* accuracy

Table 3 shows the results of the different measurements made during the PoC, identified through numbers 1 to 6 in green in Fig. 6, including the root mean squared error (RMSE) associated with each measurement. Manual measurements performed by clinical staff on preoperative and postoperative CT scans (1 and 5 measurements identified in green in Fig. 6) are used as ground-truth (GT) to conclude about the *SpineAlign* performance and are identified as “Manual” on “Preoperative” and “Postoperative” sections of Table 3. Figure 8 shows the manual assessment performed, where the superior (in red) and inferior (in blue) endplates of each vertebra are identified in the preoperative and postoperative CT scans.

Two measurements are made by *SpineAlign* after segmenting vertebrae and calculating vertebral endplates on preoperative and postoperative CT scans (2 and 6 measurements identified in green in Fig. 6). These measurements are not performed during the surgical procedure, and thus the methods developed in the scope of intraoperative spinal alignment assessment (M1 to M8) are not employed. These two measurements are identified as “System” on “Preoperative” and “Postoperative” sections of Table 3.

The preoperative RMSE when calculating the angles between the vertebrae pairs in the coronal and sagittal planes were 1.25$^{\circ }$ and 0.63$^{\circ }$, respectively. The minimum absolute error in coronal and sagittal planes in preoperative CT scan was of 0.31$^{\circ }$ (L1-L2: 1.75$^{\circ }$ vs 1.44$^{\circ }$) and 0.12$^{\circ }$ (L1-L2: 1.86$^{\circ }$ vs 1.98$^{\circ }$), respectively. On the other hand, the maximum absolute in coronal and sagittal planes in preoperative CT scan was of 1.98$^{\circ }$ (L2-L4: 0.11$^{\circ }$ vs 2.09$^{\circ }$) and 0.95$^{\circ }$ (L2-L3: 5.34$^{\circ }$ vs 6.29$^{\circ }$).

Regarding the evaluation of the spinal alignment by *SpineAlign* on postoperative CT scan, the RMSE on all vertebrae pairs was of 24.35$^{\circ }$ and 12.30$^{\circ }$ in the coronal and sagittal planes, respectively. The minimum absolute error in coronal and sagittal planes was of 8.11$^{\circ }$ (L2-L3: 10.71$^{\circ }$ vs 2.60$^{\circ }$) and 1.64$^{\circ }$ (L1-L3: 15.61$^{\circ }$ vs 17.25$^{\circ }$), respectively. On the other hand, the maximum absolute error in coronal and sagittal planes in postoperative CT scan was of 45.18$^{\circ }$ (L3-L4: 45.35 vs 0.17$^{\circ }$) and 21.80$^{\circ }$ (L1-L2: 25.80$^{\circ }$ vs 4.00$^{\circ }$).

To evaluate the intraoperative spinal alignment assessment made by *SpineAlign*, two measurements were made during the surgical procedure using the preoperative CT scan (3 and 4 measurements identified in green in Fig. 6). While one measurement is performed after vertebrae registration, the other is made at the end of the surgical procedure, with both relying on the algorithms developed in the scope of intraoperative spinal alignment assessment (M1 to M8 in Table 2). The measurement performed after vertebrae registration (3 in Fig. 6) is identified as “M1” to “M8” in “Preoperative” section of Table 3, while the measurement performed at the end of the surgical procedure (4 in Fig. 6) is identified as “M1” to “M8” in “Postoperative” section of Table 3.

For the measurement performed after vertebrae registration, the RMSE of the coronal alignment varied between 0.70$^{\circ }$ and 1.31$^{\circ }$, with M7 proving to be more accurate, while in the sagittal plane, the RMSE varied between 1.94$^{\circ }$ and 8.19$^{\circ }$, with M8 proving to be more accurate in estimating the curvature of the spine. The minimum and maximum absolute error in coronal plane when employing M7 was 0.16$^{\circ }$ (L1-L2: 1.60$^{\circ }$ vs 1.44$^{\circ }$) and 1.11$^{\circ }$ (L3-L4: 4.82$^{\circ }$ vs 3.93$^{\circ }$), respectively. On sagittal plane, M8 exhibit a minimum and maximum absolute error of 0.18$^{\circ }$ (L1-L4: 1.03$^{\circ }$ vs 1.21$^{\circ }$) and 2.66$^{\circ }$ (L1-L3: 7.25$^{\circ }$ vs 4.59$^{\circ }$), respectively.

The estimation of the postoperative spinal alignment at the end of the surgery showed an RMSE between 1.41$^{\circ }$ and 2.15$^{\circ }$ in the coronal plane among all the methods, with the best results obtained by M5 and M7. In the sagittal plane, the RMSE varied between 6.31$^{\circ }$ and 8.59$^{\circ }$, with the best results being achieved by M8. The minimum and maximum absolute error in coronal plane when employing M5 or M7 was 0.03$^{\circ }$ (L3-L4 in M7: 0.20$^{\circ }$ vs 0.17$^{\circ }$) and 2.56$^{\circ }$ (L2-L3 in M5 and M7: 0.04$^{\circ }$ vs 2.60$^{\circ }$), respectively. On sagittal plane, M8 exhibit a minimum and maximum absolute error of 0.25$^{\circ }$ (L1-L4: 19.91$^{\circ }$ vs 19.66$^{\circ }$) and 12.59$^{\circ }$ (L2-L3: 5.01$^{\circ }$ vs 17.60$^{\circ }$), respectively.

The accuracy reported in this work is based on a single PoC experiment using a porcine spine model. As such, it was not possible to perform statistical tests such as *p*-values or to compute confidence intervals due to the limited sample size. This limitation stems from the experimental protocol, which allowed only one surgical simulation under controlled conditions. Nonetheless, the results demonstrate the system’s technical feasibility and its potential to provide quantitative intraoperative feedback.

## Discussion

Regarding the clinical outcomes, in contrast to the human spine, the porcine spine used in the PoC cannot acquire any degree of lordosis. Thus, when the resection is performed at L3 in the porcine spine, the superior and inferior spine segments to the resection are straight, while in humans the spine can have some mobility that allows these segments to acquire some curvature. The consequence is that the porcine spine acquires a “V” shape in the sagittal plane, what can lead to a mismeasurement of the spine’s alignment in this plane by *SpineAlign*. This is caused because *SpineAlign* assumes that the spine will have a curved shape estimated by a Bézier curve. Nevertheless, it is important to analyze the results obtained by the *SpineAlign* in order to conclude about the consequences of the shape of the porcine in the measurements.

The measurements performed by *SpineAlign* with the preoperative and postoperative CT scans (“System” in “Preoperative;” and “Postoperative” sections of Table 3) allows to conclude if *SpineAlign* alone is capable of getting the same measurements as the clinical staff, because the methods developed in the scope of intraoperative spinal alignment assessment (spinal curve estimation, vertebrae position estimation, and vertebrae orientation correction) are not employed. Results suggest that *SpineAlign* is capable of correctly calculate the spinal alignment of the spine from the preoperative CT scan, since it was able to quantitatively assess the spinal alignment on the preoperative CT scan with small error (1.25$^{\circ }$ and 0.63$^{\circ }$ on coronal and sagittal planes, respectively). However, when using the postoperative CT scan, the vertebrae were wrongly segmented by the well established DL based algorithms (warning sign in Fig. 6). This occurred due to the different shape of human and porcine vertebrae, making the DL algorithms (trained on human vertebrae) to not perform well. Also, the osteotomy changed completely the shape of L3, what can increase the chance of errors in vertebrae segmentation. Consequently, the evaluation performed by the *SpineAlign* using the postoperative CT scan showed completely different values from the manual evaluation.

After performing the registration of vertebrae (M1 to M8 in “Preoperative” section of Table 3), *SpineAlign* was able to reproduce the preoperative alignment of the spine with high reliability and accuracy, considering that M8 showed a RMSE in estimating the alignment of vertebral pairs of 0.75$^{\circ }$ and 1.94$^{\circ }$ in coronal and sagittal planes, respectively. Regarding the measurement at the end of the surgery (M1 to M8 in “Postoperative” section of Table 3), *SpineAlign* was capable of measuring the spinal alignment with good accuracy, achieving a RMSE of 1.43$^{\circ }$ and 6.31$^{\circ }$ in the coronal and sagittal planes, respectively, when employing M8. These results shows that the error in the sagittal plane (6.31$^{\circ }$) is much higher than in the coronal plane (1.43$^{\circ }$), which can be cause by several factors, such as the “V” shape of the spine in the sagittal plane, which makes it impossible to predict this shape using a Bézier curve. In addition, the system does not take into account the changes in the intervened vertebra, what does not allow to estimate the curvature according to the planned procedure. Nevertheless, considering the intra- and inter-rater standard error of measurement (SEM) values found in the literature (0.8$^{\circ }$ to 5.0$^{\circ }$ and 2.5$^{\circ }$ to 6.2$^{\circ }$, respectively), an error of 1.43$^{\circ }$ and 6.31$^{\circ }$ in the coronal and sagittal planes, respectively, are not far from these ranges and are acceptable values [30]. Furthermore, a value of 1.43$^{\circ }$ is below the SEM inter-rater minimum, and 6.31$^{\circ }$ is close to the SEM inter-rater maximum.

Analyzing Table 3 in depth, one of the conclusions that can be drawn from the results obtained is that the method for orientation correction greatly influences the performance of the system. Methods M5 to M8 obtained better results than the others, indicating that the orientation correction by the intermediate vector ($OC_{IVN}$: Algorithm 6 and Fig. 3C) is the method with the highest accuracy. Considering that M8 was the one that obtained the best results, we can consider that the best algorithm for the curve estimation is to use the normal of the endplates and the initial positions ($CE_{ENIP}$: Algorithm 2 and Fig. 1C), while for the vertebral position estimation the best algorithm is to consider the difference in height between the tracked vertebrae ($PE_H$: Algorithm 3 and Fig. 2B).

However, since there is not much difference between M8 and M6 in terms of RMSE, the method of estimating the vertebral position by considering the perpendicular to the straight line connecting the tracked vertebrae ($PE_P$: Algorithm 4 and Fig. 2C) seems to be more robust since it is independent of the orientation of the vertebrae.

Regarding curvature estimation methods ($CE_{EN}$ and $CE_{ENIP}$), it was not possible to determine which method performs better (preoperative: 0.71$^{\circ }$ vs 0.70$^{\circ }$ and 2.30$^{\circ }$ vs 1.94$^{\circ }$ in coronal and sagittal planes, respectively; postoperative: 1.41$^{\circ }$ vs 1.41$^{\circ }$ and 7.10$^{\circ }$ vs 6.31$^{\circ }$ in coronal and sagittal planes, respectively). The small difference between both methods occurs because the segment used is composed of an even number of vertebrae (L1 to L4: 4 vertebrae). Considering Algorithms 1 and 2, they will return almost the same solution when the segment has four vertebrae, independently if the normals of the tracked vertebrae intersect or not. Only when there are more than four vertebrae in the segment, the two methods provide meaningful different results. The difference will be even greater when the number of vertebrae is even, so further PoC using a segment with a different number of vertebrae is necessary.

Currently, intraoperative spinal alignment assessment is typically performed using visual estimation through fluoroscopy or intraoperative CT. These methods either rely heavily on surgeon experience and expose patients and staff to significant radiation. Moreover, they offer limited or no quantitative evaluation of spinal curvature. In contrast, as shown in Table 4, *SpineAlign* provides real-time, quantitative feedback without any radiation exposure. While direct comparison is challenging due to the novelty of our system, these distinctions represent a clear step forward in addressing current intraoperative limitations.

**Table 4:** Qualitative comparison between *SpineAlign* and existing intraoperative spinal alignment assessment approaches

Feature	Fluoroscopy/Intraoperative CT	*SpineAlign* (proposed)
Radiation-free	No	**Yes**
Quantitative assessment	Limited	**Yes**
Real-time feedback	No	**Yes**
3D curvature estimation	Limited	**Yes**
Intraoperative usability	Moderate (setup time)	Moderate (PoC: $\approx \!$ 28 min)
Radiation exposure risk	High	**None**

*SpineAlign* offers a radiation-free and quantitative alternative, with potential to improve intraoperative decision-making

A comparative analysis between SpineAlign and recent patents is presented in Table 5. Unlike most existing solutions, which rely on extensive tracking of multiple vertebrae, preoperative implant planning, and proprietary hardware, *SpineAlign* achieves real-time curvature estimation by tracking only two vertebrae per segment. This minimalistic requirement reduces surgical burden and improves intraoperative workflow. Furthermore, while other systems typically require intraoperative imaging (fluoroscopy or CT), *SpineAlign* is entirely radiation-free. It is also agnostic to implant strategy, making it applicable to a broader range of procedures beyond deformity correction, such as decompression or minimally invasive approaches. Most notably, *SpineAlign* is the only system to explicitly model spinal curvature intraoperatively, using vertebral orientations to construct a smooth Bézier-based representation of the spinal axis. This capability provides quantitative feedback on alignment dynamics without the need for full segment exposure or complex implant-dependent navigation platforms, representing a novel and practical advancement in spinal surgery support tools.

### Limitations and future work

While *SpineAlign* demonstrates promising results as a radiation-free clinical decision support system for intraoperative spinal alignment assessment, several limitations must be acknowledged to contextualize its current stage of development.

Due to the experimental constraints of the PoC, which was performed on a single porcine spine, statistical analyses such as confidence intervals or *p*-values could not be computed. The results reported—such as a maximum RMSE of 6$^{\circ }$—should therefore be interpreted as preliminary indicators of technical feasibility.

Another source of potential error stems from the system’s reliance on tracking only two vertebrae per segment. The intermediate vertebrae are estimated using Bézier curve modeling and anatomical assumptions. While efficient, this method does not capture individual disc deformation or complex spinal mechanics, particularly after structural changes such as osteotomies.

Also, *SpineAlign* currently models the spine as a chain of rigid vertebrae, omitting the biomechanical and histological properties of intervertebral discs and surrounding soft tissues. While this simplification allows for efficient real-time feedback in the OR, it may limit clinical realism. Future versions may integrate biomechanical models or finite element simulations for enhanced assessment.

While *SpineAlign* is motivated by clinical needs in spinal surgery, it is important to emphasize that the current study represents a preclinical PoC conducted in a controlled laboratory setting. The integration of optical tracking, registration, and curvature modeling was validated on an ex vivo porcine spine to demonstrate technical feasibility. As such, the findings do not yet establish clinical efficacy or readiness for deployment in human surgeries. Rather, they lay the groundwork for future studies aimed at evaluating the system in real operative environments. By presenting the methodology in a simplified and transparent setting, we provide a reproducible foundation for further development and potential clinical translation.

The next steps in *SpineAlign*’s development will focus on conducting multi-surgeon, multi-trial studies involving different surgeons, surgical conditions, and spine types to enable robust statistical validation. Comparative studies with conventional intraoperative methods (e.g., fluoroscopy or CT) will be conducted to quantitatively evaluate the clinical benefit. Furthermore, integrating mechanical simulation may enhance the system’s intraoperative decision-making capabilities.

**Table 5:** Comparison between *SpineAlign* and existing patented systems for intraoperative spinal alignment assessment

Feature/System	*SpineAlign*	Gullotti et al. (2023) [7]	Scholl (2024) [8]	Fujita et al. (2020) [9]	Chelala et al. (2022) [10]
Tracking requirement	Only 2 vertebrae per segment; intermediate vertebrae interpolated using Bézier curve	Tracks multiple vertebrae or implanted rods	Tracks multiple vertebrae and implants	Tracks multiple vertebrae via rigid bodies fixed to spinous processes	Tracks fiducial elements affixed to vertebrae or tools
Intermediate vertebra modeling	Bézier curve interpolation	Not modeled	Not modeled	Not modeled	Not modeled
Registration method	Point- or surface-based registration using pre-op CT	Reference arrays fixed to vertebrae registered via imaging	Intraoperative CT or fluoroscopy + navigation	Optical tracking + pre-op imaging registration	Fiducial markers segmented and registered from imaging
Radiation-free	Yes - no fluoroscopy or CT during surgery	No - fluoroscopy used	No - uses intraoperative CT/fluoro	No - intraoperative imaging required	No - intraoperative imaging required
Real-time feedback	Yes - real-time curvature estimation during manipulation	Yes - intraoperative alignment checking possible	Yes - alignment monitored during procedure	Yes - updates curvature during correction	Yes - computes alignment from tracked markers
Use of preoperative implant planning	Not required - system agnostic to implant strategies	Required - rod contouring and planned corrections	Required - overlays implant plan intraoperatively	Required - pre- and post-correction comparison	Required - assumes pre-op correction targets
Dependency on surgical or tracking hardware	Requires generic optical tracking hardware (OTS + rigid bodies); no implants or proprietary navigation tools	Requires implants, reference arrays, and tools	Requires navigation system and tracked tools	Requires instrumented vertebrae with rigid trackers	Requires fiducials or instrument-mounted markers
General-purpose application	Yes - suitable for decompression, correction, or MIS	No - designed for implant-based corrective procedures	No - implant-focused	No - focused on deformity correction	No - implant-specific use
Soft tissue exposure requirement	No - only two vertebrae must be locally visible; PoC used full exposure for access	Yes - reference arrays affixed to vertebrae require exposure	Yes - visibility of hardware and bone landmarks required	Yes - rigid bodies mounted on spinous processes	Yes - fiducial markers must be visible
Focus on curvature estimation	Yes - 3D curvature modeled from endplate orientations	No - measures implant alignment only	No - navigation focus, not curvature	No - monitors displacement only	No - verifies alignment post-tracking

## Conclusion

Intraoperative assessment of a patient’s spinal alignment is currently accomplished by acquiring and interpreting CT scans or radiographs. In addition to the misleading assessment associated with this method, the amount of radiation the patient is exposed to is quite high. This work describes the CDSS developed to quantitatively assess the patient’s spinal alignment without the need for intraoperative medical imaging. A PoC was presented to evaluate the accuracy of the developed CDSS in terms of clinical applicability and overall system effectiveness. Due to its anatomical similarity and its widespread use in the validation of surgical systems, the PoC was performed on a porcine spine. The procedure was successfully performed, going from a spine without curvature to a lordotic spine, and the quantitative evaluation of the spinal alignment performed by the system could be compared with the evaluation performed manually, showing high accuracy.

Future experiments should be conducted to test the repeatability and reliability of the system, considering the following improvements: (i) redesign the references and pointer tool using a sterilizable metallic material to ensure sterilization issues and to be more robust; (ii) implement non-invasive strategies for the registration procedure using ultrasound or automatic registration if intraoperative CT is used; (iii) record the position of intermediate vertebrae and compare their position with the postoperative CT scan; (iv) consider cases with more intermediate vertebrae to conclude on the performance of the system in long segments of the spine; and (v) perform a multi-surgeon serial study.

Presently, the system is at Technology Readiness Level (TRL) 3, which corresponds to the experimental PoC described. Further steps should be considered to provide more robustness to the system, leading to a highly effective and reliable CDSS for intraoperative spinal alignment assessment. In a nutshell, the first steps towards a system capable of measuring the patient’s spinal alignment intraoperatively without the need to acquire medical images have been taken through the contribution of this work.

## Figures and Tables

**Fig. 9:**
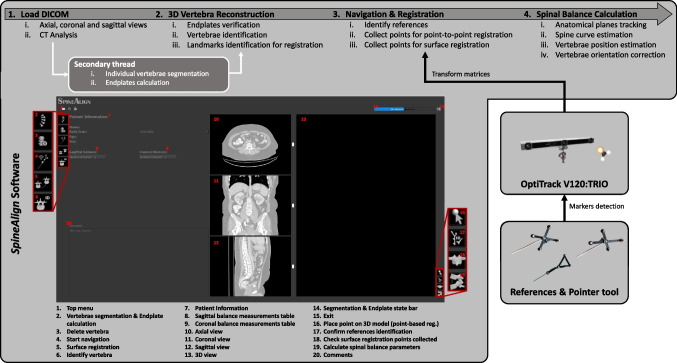
*SpineAlign* overview. Optical tracking system is integrated into the system via a NatNet server/client connection provided by the Motive software, which allows rigid body tracking in *SpineAlign*. Calculation of the patient’s spinal alignment is accomplished by following four steps described at the top of the figure

## Data Availability

The data that support the findings of this study are available from the corresponding author upon reasonable request.

## Appendix 1. SpineAlign overview and setup

*SpineAlign* (Fig. 9) is organized as a modular pipeline, illustrated through a flowchart that depicts the sequential process and data flow across the four main stages. Each stage is further supported by secondary processes such as deep learning-based segmentation, which are highlighted separately to clarify the system architecture.

*SpineAlign* relies on an OTS to provide real-time tracking of the position and orientation of surgical instruments relative to the patient’s anatomy during the surgical procedure. The *SpineAlign* framework incorporates diverse functions critical for intraoperative spinal alignment assessment. Furthermore, through a GUI, the system aids the surgeon during the surgical procedure, ultimately aiming to deliver feedback on the patient’s spinal alignment.

The system was designed to guide the surgeon along a sequence of steps as depicted at the top of Fig. 9. Drawing inspiration from the methodical protocol followed by clinical staff during CASS, four main steps are defined: (i) load DICOM; (ii) 3D vertebrae reconstruction; (iii) navigation and registration; and (iv) spinal alignment calculation. Each step performs specific tasks, as illustrated in Fig. 9. Additionally, *SpineAlign* incorporates established deep learning (DL) solutions for individual vertebrae segmentation [22, 23] and automatic endplate calculation [24] from CT scans (’Secondary thread’ in Fig. 9). These components are crucial for generating the 3D reconstruction of each vertebra and their corresponding endplates, allowing to achieve step 2 in Fig. 9 (3D Vertebra Reconstruction).

To estimate spine curvature during surgery, it is essential to monitor the movement of vertebrae, a process that corresponds to step 3 of Fig. 9 (Navigation & Registration). Surgeons accomplish this by attaching references to the vertebrae for the OTS to track. This tracking process entails calculating the position and orientation of these references using reflective markers detected by the OTS. In this work, the OptiTrack V120:TRIO was used for development purposes and interfaced with *SpineAlign* via a Nat-Net server/client connection provided by the Motive software, enabling rigid body tracking. *SpineAlign* enables the surgeon to identify the reference-vertebra pair, thereby facilitating point-to-point and surface registration of the tracked vertebrae following well-established procedures in the literature [25], which in turn enables vertebrae tracking.

## Appendix 2. Algorithms parameters

Table 6 displays the nomenclature and definition of the parameters used to describe the developed algorithms. Two examples are provided for each defined parameter. Additionally, Table 6 includes the figures’ identification indicating where the associated parameters can be found, as well as the sections where these parameters hold significance.

**Table 6:** Nomenclature and corresponding definitions of parameters used to describe the algorithms

Nomenclature	Definition	Examples	Figs.	Sections
$VR_i,\hspace{4.0pt}i=0,\ldots ,n-1$	**Identification of the** $\varvec{i^{th}}$ **tracked vertebra**, starting from the most superior ($VR_0$) to the most inferior tracked vertebra ($VR_{n-1}$)	**1.** If L1 and L5 are tracked ($n=2$): L1 is $VR_0$ and L5 is $VR_1$		
		**2.** If T2, L1, and L5 are tracked ($n=3$): T2 is $VR_0$, L1 is $VR_1$, and L5 is $VR_2$	-	2.1.1
$CM_i,\hspace{4.0pt}i=0,\ldots ,n-1$	**Center of mass (CoM) of the** $\varvec{i^{th}}$ **vertebra on a segment between two tracked vertebrae**, starting from the most superior ($CM_0$) to the most inferior tracked vertebra ($CM_{n-1}$)	**1.** If L1 and L5 are tracked, there will be 5 vertebrae in this segment - L1 to L5 ($n=5$): the CoM of L1, L2, L3, L4, and L5 are $CM_0$, $CM_1$, $CM_2$, $CM_3$, and $CM_4$, respectively		
		**2.** If T2, L1, and L5 are tracked, there will be 16 vertebrae from T2 to L5 ($n=16$). The whole segment can be divided in two smaller segments: (i) between T2 and L1 ($n=12$) and (ii) between L1 and L5 ($n=5$). Regarding the first segment, the CoM of T2 is $CM_0$, T3 is $CM_1$, and so on until L1, which has CoM $CM_{11}$. For the second segment, we follow Example 1	1 A,B,C 2 A,B,C 3 A,B,C	2.1.2 2.1.3 2.1.4
$n_{Si}$, $0\le i\le n-1$ $n_{Ii}$, $0\le i\le n-1$	**Unit vectors normal to the superior (**$\varvec{n_{Si}}$**) and inferior (**$\varvec{n_{Ii}}$**) endplates of the **$\varvec{i^{th}}$ **vertebrae with CoM** $\varvec{CM_i}$ **on a segment between two tracked vertebrae.**	**1.** If L1 and L5 are tracked, there will be 5 vertebrae in this segment - L1 to L5 ($n=5$) with CoM $CM_0$ to $CM_4$. The unit vectors normal to the superior endplates of vertebrae L1, L2, L3, L4, and L5 are defined as $n_{s0}$, $n_{s1}$, $n_{s2}$, $n_{s3}$, and $n_{s4}$, respectively. In regards to the vectors normal to the inferior endplates of L1, L2, L3, L4, and L5, they are identified as $n_{I0}$, $n_{I1}$, $n_{I2}$, $n_{I3}$, and $n_{I4}$, respectively		
		**2.** If T2, L1, and L5 are tracked, there will be 16 vertebrae from T2 to L5 ($n=16$). The whole segment can be divided in two smaller segments: (i) between T2 and L1 ($n=12$) and (ii) between L1 and L5 ($n=5$). In the first segment, T2 to L1 have corresponding CoM from $CM_0$ to $CM_{11}$. The unit vectors normal to the superior endplates of T2 is defined as $n_{S0}$, T3 is $n_{S1}$, and so on to L1 with $n_{S11}$. In regards to the inferior endplates, T2 is defined as $n_{I0}$, T3 is $n_{I1}$, and so on until L1 which is identified as $n_{I11}$. For the second segment, we follow Example 1	1 B,C 3 B	2.1.2 2.1.4
$d_{pos}$	**Euclidean distance between the CoM of the two tracked vertebrae of a segment**	**1.** If L1 and L5 are tracked, $d_{pos}$ is the Euclidean distance between the CoM of L1 and L5: $d_{pos}=||CM_0-CM_4||$		
	$$d_{pos}=||CM_0-CM_{n-1}|| $$			
		**2.** If T2, L1, and L5 are tracked, there will be 16 vertebrae from T2 to L5 ($n=16$). The whole segment can be divided in two smaller segments: (i) between T2 and L1 ($n=12$) and (ii) between L1 and L5 ($n=5$). Regarding the first segment, $d_{pos}$ is the Euclidean distance between the CoM of T2 and L1: $d_{pos}=||CM_0-CM_{11}||$. For the second segment, we follow Example 1	1 B,C2C	2.1.22.1.3
$$d_{i,j},\text { where} {\left\{ \begin{array}{ll} 0\le i\le n-2,\\ i+1\le j\le n-1 \end{array}\right. }$$	**Distance from **$\varvec{CM_i}$ to $\varvec{CM_j}$ **passing through the CoM of the vertebrae between the** $\varvec{i^{th}}$ **and** $\varvec{j^{th}}$ **vertebrae**, on a segment between two tracked vertebrae	**1.** If L1 and L5 are tracked, there will be 5 vertebrae in this segment - L1 to L5 ($n=5$). In this case, *i* can take any value between 0 and 3 ($n-2$), while *j* can take any value between $i+1$ and 4 ($n-1$). $d_{0,1}$ is the distance from $CM_0$ to $CM_1$ and is calculated as $||CM_0-CM_1||$. The same way, the distance from $CM_1$ to $CM_2$ is calculated as: $d_{1,2}=||CM_1-CM_2||$. To calculate the distance from $CM_0$ to $CM_2$ we can sum $d_{0,1}$ and $d_{1,2}$: $d_{0,2}=||CM_0-CM_1||+||CM_1-CM_2||$. The largest distance between two CoM is calculated from L1 to L5: $d_{0,4}=\sum _{k=0}^{3} ||CM_k-CM_{k+1}||=||CM_0-CM_1||+||CM_1-CM_2||+||CM_2-CM_3||+||CM_3-CM_4||$	1 A 2 A	2.1.2
	$$ d_{i,j}=\sum \limits _{k=i}^{j-1} ||CM_k-CM_{k+1}|| $$			
	$$d_{0,n-1}\ne d_{pos}$$			
		$$d_{0,4}\ne d_{pos}=||CM_0-CM_4||$$		
$\%d_i,\hspace{4.0pt}1\le i\le n-1$	**Ratio between the distance from** $\varvec{CM_0}$ **to** $\varvec{CM_i}$ **(**$\varvec{d_{0,i}}$**) and the largest distance between two CoM in the segment (**$\varvec{d_{0,n-1}}$)	**1.** If L1 and L5 are tracked, there will be 5 vertebrae in this segment - L1 to L5 ($n=5$). $\%d_0$ is 0 and corresponds to L1 with CoM $CM_0$. The largest distance in this segment is from L1 to L5 ($CM_0$ to $CM_4$): $d_{0,4}$. In this case, *i* can take any value between 1 and 4 ($n-1$). $\%d_1$ corresponds to L2 (with CoM $CM_1$) and is the ratio between $d_{0,1}=||CM_0-CM_1||$ and the largest distance in the segment: $\%d_1=d_{0,1}/d_{0,4}$. The same way, $\%d_2=d_{0,2}/d_{0,4}$, $\%d_3=d_{0,3}/d_{0,4}$, and $\%d_4=d_{0,4}/d_{0,4}=1$, corresponding to L3, L4 and L5, respectively		
	$$\%d_i=\frac{d_{0,i}}{d_{0,n-1}}$$			
	$\%d_0$ is not calculated, as it is always 0. The ratio is calculated for the vertebrae on a segment between two tracked vertebrae.	**2.** If T2, L1, and L5 are tracked, there will be 16 vertebrae from T2 to L5 ($n=16$). The whole segment can be divided in two smaller segments: (i) between T2 and L1 ($n=12$) and (ii) between L1 and L5 ($n=5$). In regards to the first segment, $\%d_0$ corresponds to T2 with CoM $CM_0$ and is 0. Also, the largest distance on the segment is from T2 to L1 ($CM_0$ to $CM_{11}$): $d_{0,11}$. *i* can take any value between 1 and 11 ($n-1$). $\%d_1$ corresponds to T3 ($CM_1$) and is the ratio between $d_{0,1}=||CM_0-CM_1||$ and the largest distance on the segment: $\%d_1=d_{0,1}/d_{0,11}$. This logic is followed for $\%d_i \text { where } 2\le i\le 11$ which are related to vertebrae T4 to L1 ($CM_3$ to $CM_{11}$), where $\%d_{11}=d_{0,11}/d_{0,11}=1$. For the second segment, we follow Example 1	-	2.1.22.1.3
$p_i, i=0,\ldots ,n-1$	**Control points of the Bézier curve that corresponds to **$\varvec{CM_i}$ **on a segment between two tracked vertebrae.**	**1.** If L1 and L5 are tracked, there will be 5 vertebrae in this segment - L1 to L5 ($n=5$): the control points corresponding to L1, L2, L3, L4, and L5 are $p_0$, $p_1$, $p_2$, $p_3$, and $p_4$, respectively. Since L1 and L5 are tracked, we know their position, so we do not need to estimate their control points: $p_0=CM_0$ and $p_4=CM_4$. The other control points, corresponding to L2, L3, and L4, have to be estimated, and for this reason $p_1\ne CM_1$, $p_2\ne CM_2$ and $p_3\ne CM_3$		
	For **tracked** vertebrae, $p_i=CM_i$ since their position is known. For **untracked** vertebrae $p_i\ne CM_i$ since $p_i$ is an estimation of $CM_i$ used to control the Bézier curve: $p_i$ controls the curve, while $CM_i$ will be a point on the curve	**2.** If T2, L1, and L5 are tracked, there will be 16 vertebrae from T2 to L5 ($n=16$). In this case, two Bézier curves are estimated: (i) between T2 and L1 ($n=12$) and (ii) between L1 and L5 ($n=5$). For the first segment (T2-L1), the control points corresponding to T2 is $p_0$, T3 is $p_1$, and down to L1 with control point $p_{11}$. In this segment, the control points corresponding to T2 and L1 are known since these vertebrae are tracked: $p_0=CM_0$ and $p_{11}=CM_{11}$. The control points corresponding to untracked vertebrae in this segment have to be estimated, and for this reason $p_i\ne CM_i,\hspace{4.0pt}\text {where } 0<i< 11$. For the second segment, we follow Example 1	1 C	2.1.2
$C(t),\hspace{4.0pt}0\le t\le 1$	**Estimated Bézier curve on a segment between two tracked vertebrae**	**1.** If L1 and L5 are tracked, *C*(*t*) is the Bézier curve estimated between these vertebrae, defined by the control points $p_0$, $p_1$, $p_2$, $p_3$, and $p_4$. The points at $t=0$ and $t=1$ correspond to the first and last control points, respectively, which are the control points corresponding to the tracked vertebrae L1 and L5 ($p_0$ and $p_4$)		
		**2.** If T2, L1, and L5 are tracked, two Bézier curves are estimated: (i) between T2 and L1 and (ii) between L1 and L5. The estimated Bézier curve *C*(*t*) on the first segment is defined by points $p_i, \text {where } 0\le i\le 11$ corresponding to vertebrae from T2 to L1. The points at $t=0$ and $t=1$ correspond to the first and last control points, respectively, which are the control points corresponding to the tracked vertebrae T2 and L1 ($p_0$ and $p_{11}$). For the second segment between L1 and L5, we follow Example 1	1 B,C	2.1.22.1.32.1.4

Examples are provided for enhanced comprehension. Figures (Figs.) correspond to the figures in which the visual perception of the parameter can be found. Sections are the sections where the parameter plays an important role

## Abbreviations

CASS: Computer-assisted spinal surgeries
CDSS: Clinical decision support system
FOV: Field-of-view
IV: Intravertebral
OR: Operating room
SD: Spinal deformities
CoM: Center of mass
CT: Computed tomography
iCT: Intraoperative CT
OTS: Optical tracking system
GUI: Graphical user interface
LAI: Left, anterior, and inferior
PoC: Proof of concept
RMSE: Root mean squared error
GT: Ground-truth
pCT: Preoperative CT
DL: Deep learning
TRL: Technology Readiness Level
MRI: Magnetic Ressonance Imaging
